# Effects of Health Insurance on Quality of Care in Low-Income Countries: A Systematic Review

**DOI:** 10.3389/phrs.2023.1605749

**Published:** 2023-08-10

**Authors:** Doris Osei Afriyie, Brendan Kwesiga, Grace Achungura, Fabrizio Tediosi, Günther Fink

**Affiliations:** ^1^ Department of Epidemiology/Public Health, Swiss Tropical and Public Health Institute (Swiss TPH), Basel, Switzerland; ^2^ University of Basel, Basel, Switzerland; ^3^ World Health Organization, Nairobi, Kenya; ^4^ World Health Organization, New Delhi, India

**Keywords:** quality of care, universal health coverage, systematic review, low-income, health insurance

## Abstract

**Objectives:** To evaluate the effectiveness of health insurance on quality of care in low-income countries (LICs).

**Methods:** We conducted a systematic review following PRISMA guidelines. We searched seven databases for studies published between 2010 and August 2022. We included studies that evaluated the effects of health insurance on quality of care in LICs using randomized experiments or quasi-experimental study designs. Study outcomes were classified using the Donabedian framework.

**Results:** We included 15 studies out of the 6,129 identified. Available evidence seems to suggest that health insurance has limited effects on structural quality, and its effects on the process of care remain mixed. At the population level, health insurance is linked to improved anthropometric measures for children and biomarkers such as blood pressure and hemoglobin levels.

**Conclusion:** Based on the currently available evidence, it appears that health insurance in LICs has limited effects on the quality of care. Further studies are required to delve into the mechanisms that underlie the impact of health insurance on the quality of care and identify the most effective strategies to ensure quality within insurance programs.

**Systematic Review Registration:** https://www.crd.york.ac.uk/prospero/display_record.php?RecordID=219984, identifier PROSPERO CRD42020219984

## Introduction

In the past decades, many low-income and lower-middle income countries (LLMICs) have made commitments to make progress towards universal health coverage (UHC), a critical component of the sustainable development goals (SDGs) [1]. UHC aims to ensure that all people have equitable access to quality essential health services without financial hardship [2]. To accelerate progress towards this goal, many LLMICs have invested in health insurance [3].

Countries have implemented an array of health insurance schemes consisting of both mandatory and voluntary schemes. Traditional social health insurance pools low and high-risk individuals who contribute a compulsory premium—typically a fixed percentage of their salaries to these schemes. In countries such as Kenya, Tanzania, and Cambodia, social health insurance targets civil servants, and formally employed workers. In order to reach households in the informal sector, countries such as Burkina Faso, India, Nepal, and Senegal have introduced voluntary schemes such as community-based health insurance (CBHIs) or mutual health insurance schemes. Some countries have established more than one type of insurance schemes for either formal or informal sectors. Tanzania, for example, has National Health Insurance Fund (NHIF) for the formal sector and offers the improved community health fund (iCHF) for the informal sector. In practice, countries such as Gabon, Ghana, Kenya and Zambia have mixed national health insurance schemes, which pool both formal and informal sector contributions.

There is growing literature on the impact of health insurance schemes on specific UHC goals. Five out of six systematic reviews published between 2012 and 2020 found strong evidence that health insurance schemes improved the use of health services [4–7]. Four systematic reviews also examined the effect of health insurance schemes on financial protection, finding mixed evidence [4, 7–9]. In this manuscript, we focus on the impact of health insurance on quality of care. Given the attention on coverage and financial risk reduction, the impact of insurance on quality is not obvious and can potentially be negative if supply-side factors are not adjusted to match the additional demand created by insurance coverage. Furthermore, given the critical importance of high quality of care for improving health outcomes in low-income setting [10], investigating the impact of insurance on quality and the mechanisms resulting in this effect is of high importance for the current and future rollout of insurance programs.

### Conceptualization of Quality of Care-Donabedian Framework

Several frameworks have been developed to measure quality of care. We use the Donabedian model, which has been used widely in the literature to define quality of care, here [11]. The framework defines quality along three main dimensions: structure, process and outcomes of care [12]. Structural quality comprises of the physical and organizational characteristics in health facilities that support and steer the provision of care. Process of care assesses the technical quality of care such as appropriateness of treatment, competence in diagnostic and therapeutic procedures. Process of care also includes interpersonal care, which assess the social and psychological interaction between providers and patients. Finally, outcomes of care include the effects of care on individuals and populations, changes to health status, patient satisfaction and health-related quality of life. The framework is summarized with examples for each domain in Table 1.

**TABLE 1:** Donabedian Framework on quality of care (Health Insurance and Quality of Care in low-income countries, 2010–2022).

Quality domain	Description of domain	Examples of indicators used
Structural	Physical and organizational characteristics of the facility or practice where healthcare occurs	Quality of physical infrastructure, availability of drugs and medical supplies
Process: Technical	Providers’ activities in delivering care	Content of care (correct diagnosis, appropriate treatment, physical examination, Counselling), Prescription practices
Process: Interpersonal care	Patients’ subjective experiences not directly related to the clinical care received	Patient perception (waiting time, communication, confidentiality, Attitudes of health providers, sufficient time spent with provider)
Outcome	Effects of care on health status of individuals and populations	Mortality rates, patient-reported health measures, anthropometric measures, overall patient satisfaction

Studies have shown that health insurance schemes use a mix of strategies to empower patients and improve provider performance [13, 14]. Some schemes use regulations such as accreditations, standard treatment guidelines and audits, to ensure enlisted providers are competent and can provide quality services. Providers that adhere to these regulations receive incentives from insurance agencies, which can be additional resources to improve the structural elements of health facilities for a higher quality of care. Furthermore, through the freedom of choice to select providers, members can “exit” from low-quality health providers and incentivize providers to maintain or improve the quality of their services [13]. Despite the rationale, there is limited systematic evidence of the effectiveness of these strategies by health insurance programs to influence quality of care. The last review dates back to 2011, finding only limited evidence of links between health insurance and quality of care in LLMICs [8]. The objective of this study is thus to systematically review the more recent evidence on the links between health insurance schemes and quality of care within LLMICs.

## Methods

### Search Strategy

We followed the Preferred Reporting Items for Systematic Review and Meta-analysis (PRISMA) protocol guidelines. The protocol for the study was registered in advance in PROSPERO as CRD42020219984. A comprehensive search of peer-reviewed and grey-literature was conducted using seven electronic databases (Medline, Embase, EconLit, PyscInfo, Web of Science, COCHRANE Central Registry of Trials and WHO Global Index Medicus) for studies published between January 2010 and August 2022. We searched both MeSH terms and keywords related to health insurance schemes and quality of care. An example of full search terms used for Medline and Embase databases can be found in Supplementary Appendix S1. We also searched the reference lists of all studies that met the inclusion criteria and other similar systematic reviews to identify further relevant articles. Authors of articles that were inaccessible were contacted to obtain full text version of their respective papers.

### Study Selection

We included empirical research reporting randomized experiments and quasi-experimental designs that assessed the effects of health insurance schemes and any of the Donabedian quality indicators in low-income countries. The review included studies published in English, which reported on public (national health insurance, social health insurance and community-based/mutual health insurance) and private health insurance schemes.

There are notable distinctions between the implementation of health insurance programs in low-income countries and high-income countries, particularly regarding fund collection and coverage. Moreover, low-income countries face the greatest challenges in terms of providing adequate quality of care [10]. Consequently, the issues related to health insurance and quality of care in low-income countries, can significantly diverse from those encountered in high-income countries. This review specifically concentrates on assessing the quality of care in low-income countries. In this study, we defined low-income countries as those classified by the World Bank as either low-income or lower-middle income in 2022. We excluded longitudinal cohort, case-control, cross-sectional studies, qualitative studies, policy briefs, commentaries, conference abstracts and editorials.

After duplicates were removed, two authors (DOA and BK) independently conducted an initial screening of titles and abstracts using the specified inclusion criteria. Non-agreement was resolved through discussion between the two authors. We then retrieved the full text of articles that met or possibly met the criteria. Again, DOA and BK independently checked the full text articles based on the inclusion/exclusion criteria for studies, and non-agreement was resolved through discussions with the other authors.

### Data Extraction and Data Analysis

For all relevant studies, a standardized data extraction form was developed. Two authors independently extracted the necessary information from studies, and any differences in data extracted were discussed and resolved. For each of the study, we extracted information on study design, name and type of health insurance, sources of data and study populations. We also extracted information on whether schemes were accompanied by any quality assurance initiatives to ensure compliance of empaneled health facilities with the standards of quality set by the health insurance or quality improvement programs to enhance the quality of care provided in health facilities [15]. Additionally, we extracted outcome(s) and main findings including descriptive statistics, point estimates and confidence intervals if available. The outcomes were grouped according to the Donabedian framework-structural, process (technical and interpersonal care) and outcome.

Two authors independently assessed the risk of bias of included studies using the appropriate tool. For randomized control trials, we applied the COCHRANE Risk-Bias tool for randomized trials [16]. For non-randomized designs, we used the Risk of Bias in Non-randomized studies of interventions (ROBINS-1) tool [17]. The COCHRANE Risk Bias tool assesses bias across five domains (randomization, deviation from intended intervention, missing outcome data, measurement of outcome, and selection of reported results) while ROBINS-1 assess bias across seven domains (confounding, selection of participants, deviations from intended interventions, missing data, measurement of outcomes and selection of reported results). The overall risk bias of each study was categorized as “high,” “moderate” or “low.” Discrepancies in assessments were resolved through consensus. As we selected studies with rigorous study designs, all studies were included in the analysis regardless of its risk of bias category.

We synthesized the findings from included studies by narrative synthesis using the Donabedian classification of its outcome [structural, process (technical and interpersonal care) and outcome].

## Results

**FIGURE 1:**
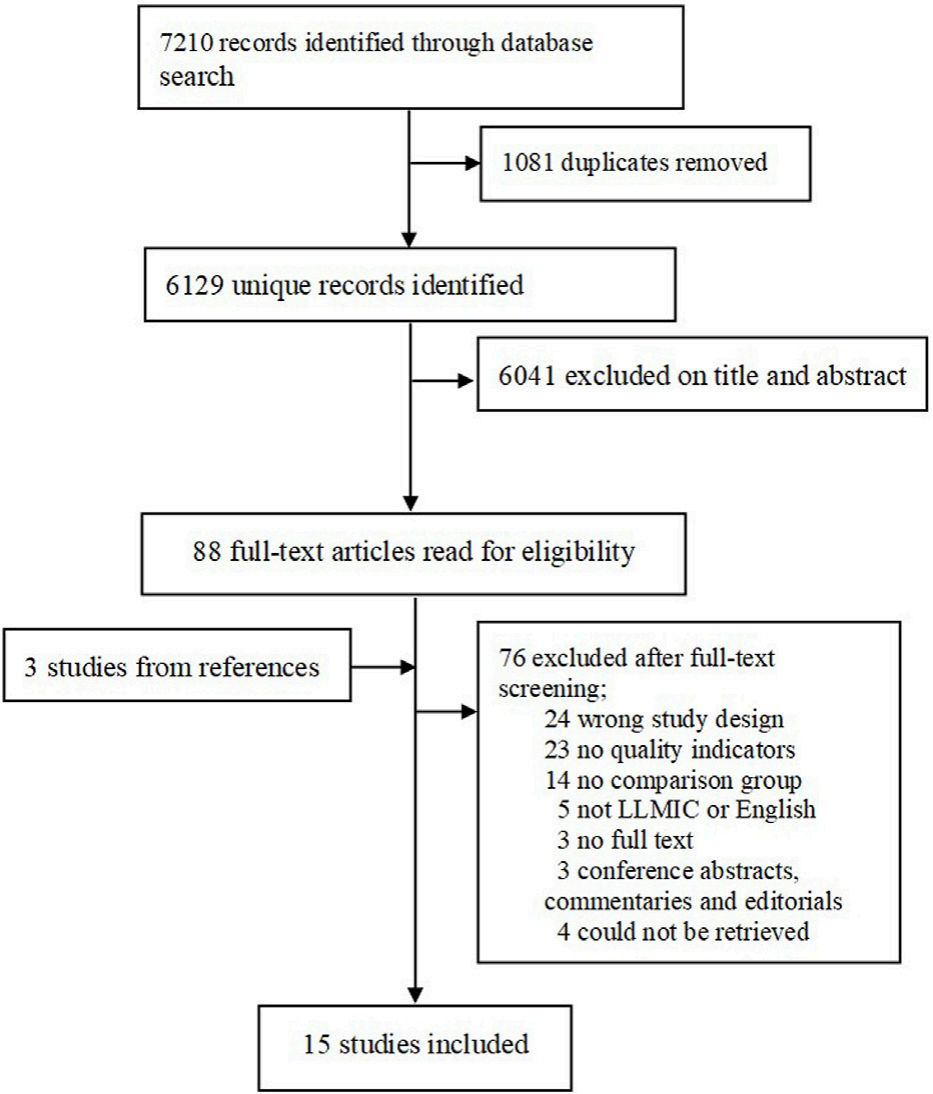
Flow chart of included studies (Health insurance and Quality of care in low-income countries, 2010–2022).

Our search strategy identified 6,129 unique records of which 6,041 did not meet the inclusion criteria. A total of 88 records were screened for eligibility by full-text review. An additional 76 articles were excluded due to various reasons such as inappropriate study designs (*n* = 38) and no quality of care indicators (*n* = 23). A total of 15 studies were included in our final review from 11 countries in Sub-Saharan Africa and Asia (Figure 1). The characteristics of the included studies are shown in Tables 2, 3. Each of the studies evaluated schemes from a single country. Three studies were conducted in Ghana, two studies each in Nigeria, and Vietnam and one study each from Burkina Faso, Ethiopia, India, Mauritania, Philippines, Uganda, and Tanzania. Out of the 14 studies which reported the years of the scheme’s implementation and time period of data analysis, half (*n* = 7) assessed data 1–3 years after the scheme’s implementation, 4 studies for 4–7 years and 2 studies for 8 years or more. The most common source of data was private survey-survey by researchers (*n* = 8) followed by public or government household survey (*n* = 6). Among the 15 studies, 47 quality of care indicators were evaluated as study outcomes. The most common Donabedian quality of care dimension that studies evaluated was the outcome domain (*n* = 19) followed by structural (*n* = 14), process-interpersonal (*n* = 11), and process-technical dimension (*n* = 3).

**TABLE 2:** Characteristics of included studies in the systematic review (Health insurance and Quality of care in low-income countries, 2010–2022).

Author (Year)	Country	Data sources	Empirical methods/approach	Health insurance	Sample size	Bias	Outcome measured
Asuming (2013)	Ghana	Household survey	Randomized experiment	Wa West District MHI (voluntary)	4,625 individuals	Low	Sick days; Performance of daily activities
Bagnoli (2019)	Ghana	MICS 2011	Propensity-score matching	NHIS (voluntary and mandatory)	7,092 children	Moderate	Stunting, anemia
Fink (2013)	Burkina Faso	HDSS Household Survey 2003–2008	Stepped wedge cluster randomization	Nouna CBHI (voluntary)	22,708 individuals-(Mortality), 1,128–1,143^b^ individuals (Perceived quality of care	Low	Age-group specific mortality, Perceived quality of care (facility hours, equipment adequacy, room adequacy, facility hygiene and staff availability
Hendricks (2014)	Nigeria	Household survey	Difference-in-differences	Hygeia Community Healthcare program (voluntary)	336 individuals	Moderate	Blood pressure
Hendricks (2016)	Nigeria	Household survey	Difference-in-differences	Kwara State Health Insurance^a^ (voluntary)	413 individuals	Moderate	Blood pressure
Jafree (2021)	Pakistan	Individual survey	Propensity score matching	MHI (voluntary)	413 individuals	Moderate	Perceived overall health
Kuwawenaruwa (2019)	Tanzania	Health facility assessment; Household survey	Difference-in-differences	NHIF’s KfW scheme (non-contributory)	49 health facilities, 4,949 women	Moderate	Drug supply, availability of contraceptives, availability of medical supplies, facility quality, functionality of equipment, interpersonal care for ANC, content for care for ANC care, content of care for PNC, experience of ANC, waiting time
Lambon-Quayefio (2017)	Ghana	DHS 2014	Propensity-score matching	Ghana NHIS (voluntary and mandatory)	23,118 children	Moderate	Neonatal mortality
Nguyen (2020)	Vietnam	LSS 2002, 2004, 2006	Triple-differences; difference-in-discontinuities	Public health insurance (non-contributory for children under 6)	26,626 children	Moderate	Days in bed, Days with limited activity
Nguyen (2019)	Vietnam	LSS 2002, 2004,2006, 2008	Difference-in-differences	Public health insurance (non-contributory for children under 6)	16,936 children	Moderate	Sick days
Nshakira-Rukundo (2020)	Uganda	Household survey	Instrumental variable	Kisiizi CHBI (voluntary)	458 individuals	Moderate	Stunting
Philibert (2017)	Mauritania	DHS 2001; NSIMM 2003; MICS in 2007, 2011	Difference- in differences	Obstetric Risk Insurance (voluntary)	25,693 women	Moderate	Neonatal mortality
Quimbo (2011)	Philippines	Patient exit survey	Randomized experiment	PhilHealth (voluntary)	941–984^b^ children	Low	CRP, wasting
Shigute (2020)	Ethiopia	Health facility assessment; Household survey	Difference-in-differences	CBHI (voluntary)	48 health lefts, 1,156 individuals	Moderate	Revenue, drug availability, equipment availability, water supply, electricity access, shortage of budget, shortage of drugs, waiting time, patient satisfaction
Sood (2016)	India	Household survey	Geographic regression discontinuity	VAS (non-contributory)	6,964 households		Post operation wellbeing (self-care, usual activities, walking ability, pain, anxiety, overall health), post operation infections, rehospitalization rates

^a^ Formerly known as Hygeia Community Healthcare program, CBHI: community based health insurance; DHS: demographic and health survey; HDSS: health and demographic surveillance site; LSS: living standard survey; MICS: multiple indicator cluster survey; NSIMM: national survey on infant mortality and malaria; NHIS: national health insurance scheme; RCT: randomized control trial; VAS: Vajpayee Arogyashree scheme.

^b^ A range was reported as number of missing variables vary by variable.

**TABLE 3:** Summary of selected characteristics of included studies in the systematic review (Health Insurance and Quality of care in low-income countries, 2010–2022).

Characteristics	Number of studies
Data source	
Public or government household survey^a^	6
Health facility assessment	2
Patient exit survey	1
Private survey^b^	8
Total	17^c^
Type of Health Insurance Scheme	
Voluntary and mandatory	1
Voluntary	9
Non-contributory	3
Total	13^d^
Post-establishment years analyzed	
1–3	7
4–7	4
≥8	3
Total	14^e^
Quality of care indicator analyzed	
Structural	14
Process-technical	3
Process-interpersonal	11
Outcome	19
Total	47

^a^ Including Multiple Indicator Cluster Survey, Living Standard Survey, and Demographic Health Survey.

^b^ Including household and individual surveys conducted by researchers.

^c^ Some studies used multiple sources.

^d^ Some schemes were analyzed by more than one study.

^e^ One study did not report years.

Three studies were randomized experiments, and the remaining studies (*n* = 12) used quasi-experimental designs. Studies used quasi-experimental designs such as difference-in-differences analysis (*n* = 7), propensity score matching (*n* = 3), instrumental variable (*n* = 1) and geographic regression discontinuity (*n* = 1).

Using the Cochrane risk of bias tool for randomized studies, the overall rating for the risk of bias was low. Among the non-randomized studies, the overall rating for all studies was moderate based on the ROBINS- I tool.

### Structural Quality Dimension

Two studies from Tanzania and Ethiopia reported on several structural quality indicators including the availability of drugs, medical supplies and the functionality of amenities (Supplementary Appendix S2) [18, 19]. Both studies reported positive effects for many of the indicators, but only three out of the 14 indicators showed significant improvements.

### Process Dimension: Technical

One study examined the impact of a scheme for pregnant women in Tanzania on three technical quality measures and reported significant improvement for only postnatal care for mothers (Supplementary Appendix S3) [18]. They reported no change for the overall PNC for infants or the ANC whether it was measured through observation of patient-provider interaction or household survey with patients [18].

### Process Dimension: Interpersonal Care

Three studies reported the effect of health insurance schemes on interpersonal care (Supplementary Appendix S3) [18–20]. One study from Tanzania found that health insurance was associated with improved scores on an index of interpersonal care for postnatal services based on 13 items [18]. Two studies reported no effect on waiting times to receive health services [18, 19]. One of the two studies found that in intervention areas, long waiting times significantly reduced the proportion of women for ANC visits [18]. A study from rural Burkina Faso reported that a CBHI rollout significantly declined half of quality of care ratings [20].

### Outcome Dimension

Only one study evaluated overall patient satisfaction and reported a positive association (Supplementary Appendix S4) [19]. Five studies assessed self-reported health outcomes (Supplementary Appendix S4) [21–25]. Three studies reported significant improvements in some of the outcome measures assessed [21, 23, 25]. Two studies assessed only one outcome measure and one reported a positive improvement while the other reported no significant effect [22, 24].

Three studies evaluated the effects of health insurance on age-specific mortality rates [20, 26, 27]. One study from Ghana, reported a positive improvement in neonatal mortality after the National Health Insurance Scheme (NHIS) [26]. Two studies from Mauritania and rural Burkina Faso, reported no significant effect on neontatal mortality and under-five mortality, respectively [20, 27]. The study in n rural Burkina Faso, also reported an increased mortality for individuals aged 65 and older [20].

Three studies reported positive effects on different anthropometric measures for children under-five. In Ghana, NHIS was found to positively impact on the height-for-age score, but the gains were not shared equally across regions with lower quality of care [28]. Nshakira-Rukundo et al, found that enrolment in a CBHI in rural Uganda was associated with a 4.3% percentage point less probability of stunting [29]. In the Philippines, health insurance decreased the likelihood of wasting among children by 9–12 percentage points [30].

Three studies evaluated the impact of health insurance on biomarkers and found positive results. In Ghana, NHIS significantly was found to reduce the probability of anemia among children by 20% [28]. In rural Nigeria, CBHI was associated with a significant decrease in blood pressure two and 4 years post-implementation [31, 32]. In the Philippines, health insurance was found to also reduce the likelihood of an infection by 4–9 percentage points among children [30].

## Discussion

This study sought to systematically review the impact of health insurance schemes on the quality of care in LLMICs. We identified 15 studies in 11 countries that rigorously evaluated the effects of health insurance schemes on diverse quality of care indicators. We found a large number of studies overall, but only a small number of studies meeting high quality evidence criteria. The findings of this study indicate that the impact of health insurance in LLMICs on quality care is not clearly established. While there were some beneficial effects of health insurance on structural quality indicators, the evidence regarding the impact on the process of care is inconclusive. Additionally, the relationship between health insurance schemes and mortality rates is varied and inconclusive. However, there was a strong positive effect on anthropometric measures for children and biomarkers such as blood pressure, C-reactive protein and hemoglobin levels.

Only two studies measured structural quality in health facilities after introducing health insurance schemes. Given the persistent challenge of the structural quality of care in many low-income countries and the rationalization to use health insurance schemes to increase revenue for health facilities to improve these challenges, evidence gaps appear particularly scarce. Both studies generally found positive results, however nearly two-thirds of indicators that they measured did not show significant improvements. The absence of statistically significant results may be due to the small number of observations (particularly at health facility level) in the two studies and indicators assessed. The results could also be potentially be the absence of an effect of insurance in improving structural care based on previous findings from Tanzania and Ethiopia. Qualitative studies from both countries have found that low reimbursement rates [18, 33–36] and reimbursement delays by health insurance authorities lead to financial constraints at health facilities to improve the drug and medical supplies challenges health facilities are already facing [33, 36, 37].

Our review also found limited evidence on health insurance improving processes of care. Only one relevant study examined technical quality after the introduction of a health insurance scheme and found that health insurance was associated with improvement of one content of care indicator. The insurance scheme may have improved specific indicators if financial incentives to providers targeted specifically those indicators [38]. A small number of studies also examined patient waiting times and found no effect of health insurance. This finding is inconsistent with the systematic review by Spaan et al, which found that health insurance schemes shorten waiting times [8]. Furthermore, only one study examined the perceived quality of care, finding negative effects. Although subjective experiences and perceptions of care are crucial for enrolment and retention rates [39–41], many of the schemes in low-income countries rarely consider patient experiences as part of health facilities maintaining their accreditation status or quality improvement measures. Health insurance authorities may consider approaches to integrate patient experiences into the accreditation of health facilities or quality improvement initiatives [42, 43].

Improving the health status and wellbeing of populations are the ultimate goals of any health system. Our finding that the effect of health insurance on mortality is mixed departs from recent studies from high-income countries [44–46]. Given that on average quality of care is poor in both the public and private sectors [47], simply increasing access to health facilities without the appropriate provider incentives will likely lead to no significant changes in health outcomes. In Burkina Faso, the negative effects of its CBHI on mortality appeared to have been driven by the adverse provider incentives that resulted in the decline of the quality of care [20]. It is also possible that it will take longer and larger sample sizes to see the true health impact of health insurance schemes in these settings. Studies in our review assessed mortality over short periods. Larger population-level studies over a longer period are ultimately needed to address this. In contrast to the negative mortality effects observed in Burkina, health insurance programs in India, rural Nigeria and Philippines were associated with improved health outcomes such as post hospitalization wellbeing, blood pressure, reducing wasting and C-reactive protein levels. These programs appear to have been coupled with supply-side interventions to address quality of care issues such as the empanelment of high-quality health facilities, upgrading of health facilities and the training of health workers and provision of financial incentives to providers to deliver high-quality care [21, 31, 32, 48, 49]. This finding suggests that addressing supply-side factors are essential to improving health outcomes. Studies also reported that health insurance was associated with better anthropometric measures for children under-five. We suspect that the improvement in anthropometric measures was driven mainly by increase in access of care rather than improvements in quality. This is inconsistent with a systematic review which found mixed results for health outcomes among children [49].

This study provides a comprehensive systematic review of health insurance schemes on the quality of care in low-income countries. The strengths of this study include the use of the Donabedian model in conceptualizing quality of care. However, the results should be interpreted carefully in light of some limitations. First, we included only studies published in English and therefore excluded other languages in our search strategy. Second, most of the studies did not investigate the length of enrolment into insurance schemes, which may how health insurance affect quality of care. In light of the limitations of the included studies, robust studies are needed to examine the causal impact of health insurance schemes particularly for process indicators such as appropriate treatment, diagnosis and patients’ experiences of care. It is also important for studies to explore the actual causal pathways that health insurance schemes in low-income countries can affect providers’ behaviors. In addition, understanding the contextual factors surrounding the health insurance is important to determine how and why these factors influence the ability of insurance schemes to affect quality of care.

In conclusion, this systematic review suggests that health insurance schemes in low -income countries have limited effects on quality of care. If the expectation of health insurance schemes is to provide additional resources to address quality of care challenges, our findings suggest they do not so. Furthermore, if health insurances schemes were designed to change providers’ behavior to improve processes of care, our findings shows that there is little impact. Our findings can serve as a resource to countries considering the use of health insurance schemes to improve quality of care.
