# Supplementary material for: Effects of Health Insurance on Quality of Care in Low-Income Countries: A Systematic Review
Source: Public Health Rev. 2023 Aug 10;44:1605749. doi: 10.3389/phrs.2023.1605749 (PMC10447888; doi:10.3389/phrs.2023.1605749)
Supplement: Supplementary file 1 [file DataSheet1.docx]

**Appendix 1: Search strategy**

**Ovid MEDLINE(R) ALL <1946 to August 19, 2022**>**> Searched 21 August 2022**

--------------------------------------------------------------------------------

1 Developing Countries.sh,kf. (91613)

2 Africa/ or Asia/ or Caribbean/ or West Indies/ or Middle East/ or South America/ or Latin America/ or Central America/ (91000)

3 (Africa or Asia or Caribbean or West Indies or Middle East or South America or Latin America or Central America).tw. (243214)

4 (Afghanistan or Albania or Algeria or Angola or Argentina or Armenia or Armenian or Azerbaijan or Bangladesh or Benin or Byelarus or Byelorussian or Belarus or Belorussian or Belorussia or Belize or Bhutan or Bolivia or Bosnia or Herzegovina or Hercegovina or Botswana or Brazil or Bulgaria or Burkina Faso or Burkina Fasso or Upper Volta or Burundi or Urundi or Cambodia or Khmer Republic or Kampuchea or Cameroon or Cameroons or Cameron or Camerons or Cape Verde or Central African Republic or Chad or China or Colombia or Comoros or Comoro Islands or Comores or Mayotte or Congo or Zaire or Costa Rica or Cote d'Ivoire or Ivory Coast or Cuba or Djibouti or French Somaliland or Dominica or Dominican Republic or East Timor or East Timur or Timor Leste or Ecuador or Egypt or United Arab Republic or El Salvador or Eritrea or Ethiopia or Fiji or Gabon or Gabonese Republic or Gambia or Gaza or Georgia Republic or Georgian Republic or Ghana or Grenada or Guatemala or Guinea or Guiana or Guyana or Haiti or Honduras or India or Maldives or Indonesia or Iran or Iraq or Jamaica or Jordan or Kazakhstan or Kazakh or Kenya or Kiribati or Korea or Kosovo or Kyrgyzstan or Kirghizia or Kyrgyz Republic or Kirghiz or Kirgizstan or Lao PDR or Laos or Lebanon or Lesotho or Basutoland or Liberia or Libya or Macedonia or Madagascar or Malagasy Republic or Malaysia or Malaya or Malay or Sabah or Sarawak or Malawi or Mali or Marshall Islands or Mauritania or Mauritius or Agalega Islands or Mexico or Micronesia or Middle East or Moldova or Moldovia or Moldovian or Mongolia or Montenegro or Morocco or Ifni or Mozambique or Myanmar or Myanma or Burma or Namibia or Nepal or Netherlands Antilles or Nicaragua or Niger or Nigeria or Muscat or Pakistan or Palau or Palestine or Panama or Paraguay or Peru or Philippines or Philipines or Phillipines or Phillippines or Papua New Guinea or Romania or Rumania or Roumania or Rwanda or Ruanda or Saint Lucia or St Lucia or Saint Vincent or St Vincent or Grenadines or Samoa or Samoan Islands or Navigator Island or Navigator Islands or Sao Tome or Senegal or Serbia or Montenegro or Seychelles or Sierra Leone or Sri Lanka or Solomon Islands or Somalia or Sudan or Suriname or Surinam or Swaziland or South Africa or Syria or Tajikistan or Tadzhikistan or Tadjikistan or Tadzhik or Tanzania or Thailand or Togo or Togolese Republic or Tonga or Tunisia or Turkey or Turkmenistan or Turkmen or Uganda or Ukraine or Uzbekistan or Uzbek or Vanuatu or New Hebrides or Venezuela or Vietnam or Viet Nam or West Bank or Yemen or Zambia or Zimbabwe).tw. (1319988)

5 exp africa/ or algeria/ or egypt/ or libya/ or morocco/ or tunisia/ or cameroon/ or central african republic/ or chad/ or congo/ or "democratic republic of the congo"/ or equatorial guinea/ or gabon/ or burundi/ or djibouti/ or eritrea/ or ethiopia/ or kenya/ or rwanda/ or somalia/ or south sudan/ or sudan/ or tanzania/ or uganda/ or angola/ or botswana/ or lesotho/ or malawi/ or mozambique/ or namibia/ or south africa/ or swaziland/ or zambia/ or zimbabwe/ or benin/ or burkina faso/ or cape verde/ or cote d'ivoire/ or gambia/ or ghana/ or guinea/ or guinea-bissau/ or liberia/ or mali/ or mauritania/ or niger/ or nigeria/ or senegal/ or sierra leone/ or togo/ or americas/ or exp caribbean region/ or exp west indies/ or exp central america/ or belize/ or costa rica/ or el salvador/ or guatemala/ or honduras/ or nicaragua/ or panama/ or panama canal zone/ or latin america/ or mexico/ or exp south america/ or argentina/ or bolivia/ or brazil/ or chile/ or colombia/ or ecuador/ or french guiana/ or guyana/ or paraguay/ or peru/ or suriname/ or uruguay/ or venezuela/ or asia/ or asia, central/ or kazakhstan/ or kyrgyzstan/ or tajikistan/ or turkmenistan/ or uzbekistan/ or exp asia, southeastern/ or borneo/ or brunei/ or cambodia/ or timor-leste/ or indonesia/ or laos/ or malaysia/ or mekong valley/ or myanmar/ or philippines/ or singapore/ or thailand/ or vietnam/ or asia, western/ or bangladesh/ or bhutan/ or india/ or sikkim/ or middle east/ or afghanistan/ or bahrain/ or iran/ or iraq/ or israel/ or jordan/ or kuwait/ or lebanon/ or oman/ or qatar/ or saudi arabia/ or syria/ or turkey/ or united arab emirates/ or yemen/ or nepal/ or pakistan/ or sri lanka/ or far east/ or china/ or beijing/ or macau/ or tibet/ or korea/ or mongolia/ or taiwan/ or indian ocean islands/ or comoros/ or madagascar/ or mauritius/ or reunion/ or seychelles/ or pacific islands/ or exp melanesia/ or exp micronesia/ or polynesia/ or pitcairn island/ or exp samoa/ or tonga/ or prince edward island/ or west indies/ or "antigua and barbuda"/ or bahamas/ or barbados/ or cuba/ or dominica/ or dominican republic/ or grenada/ or guadeloupe/ or haiti/ or jamaica/ or martinique/ or netherlands antilles/ or puerto rico/ or "saint kitts and nevis"/ or saint lucia/ or "saint vincent and the grenadines"/ or "trinidad and tobago"/ or united states virgin islands/ or oceania/ (1332086)

6 ((developing or less* developed or under developed or underdeveloped or middle income or low* income or underserved or under served or deprived or poor*) adj (countr* or nation? or population? or world or state*)).ti,ab. (125135)

7 ((developing or less* developed or under developed or underdeveloped or middle income or low* income) adj (economy or economies)).ti,ab. (848)

8 (low* adj (gdp or gnp or gross domestic or gross national)).tw. (319)

9 (low adj3 middle adj3 countr*).tw. (26034)

10 (lmic or lmics or third world or lami countr*).tw. (11238)

11 transitional countr*.tw. (175)

12 or/1-11 (2053135)

13 exp Insurance, Health/ (156805)

14 (health adj2 insur*).ti,ab,kw. (51584)

15 or/13-14 (192047)

16 "quality of health care"/ or "outcome and process assessment, health care"/ or outcome assessment, health care/ or patient outcome assessment/ or critical care outcomes/ or patient reported outcome measures/ or treatment outcome/ or process assessment, health care/ or peer review, health care/ or program evaluation/ or benchmarking/ or quality assurance, health care/ or total quality management/ or quality improvement/ or value-based health insurance/ or quality indicators, health care/ or "standard of care"/ or "utilization review"/ or patient satisfaction/ or patient preference/ or needs assessment/ or evaluation study/ or waiting lists/ or checklist/ (1787737)

17 ((qualit* adj3 (health or healthcare or treatment* or outcome* or manag* or assur* or improv* or indicator* or standard* or assess* or evaluat* or benchmark*)) or (patient* adj2 satisfact*) or (waiting adj2 (time* or list*)) or compliance or performance or checklist*).ti,ab,kw. (1846755)

18 or/16-17 (3364125)

19 12 and 15 and 18 (3462)

20 limit 19 to yr="2020 -Current" (696)

***************************

**Embase <1974 to 2022 August 19>**

Date searched: 21 August 2022

--------------------------------------------------------------------------------

1 health insurance/ or child health insurance/ or community-based health insurance/ or health insurance eligibility/ or national health insurance/ or private health insurance/ or public health insurance/ or universal health insurance/ (154959)

2 ((health* or cover*) adj2 insur*).ti,ab,kw. (81495)

3 or/1-2 (187369)

4 quality control/ or quality control procedures/ or benchmarking/ or clinical audit/ or nursing audit/ or total quality management/ or quality improvement study/ or health care quality/ or performance measurement system/ or program evaluation/ or evaluation study/ or program effectiveness/ or "utilization review"/ or patient satisfaction/ or patient-reported outcome/ or checklist/ or needs assessment/ or outcome assessment/ (1545212)

5 ((qualit* adj3 (health or healthcare or treatment* or outcome* or manag* or assur* or improv* or indicator* or standard* or assess* or evaluat* or benchmark*)) or (patient* adj2 satisfact*) or (waiting adj2 (time* or list*)) or compliance or performance or checklist*).ti,ab,kw. (2373429)

6 or/4-5 (3521147)

7 (afghanistan or albania or algeria or american samoa or angola or "antigua and barbuda" or antigua or barbuda or argentina or armenia or armenian or aruba or azerbaijan or bahrain or bangladesh or barbados or republic of belarus or belarus or byelarus or belorussia or byelorussian or belize or british honduras or benin or dahomey or bhutan or bolivia or "bosnia and herzegovina" or bosnia or herzegovina or botswana or bechuanaland or brazil or brasil or bulgaria or burkina faso or burkina fasso or upper volta or burundi or urundi or cabo verde or cape verde or cambodia or kampuchea or khmer republic or cameroon or cameron or cameroun or central african republic or ubangi shari or chad or chile or china or colombia or comoros or comoro islands or iles comores or mayotte or democratic republic of the congo or democratic republic congo or congo or zaire or costa rica or "cote d’ivoire" or "cote d’ ivoire" or cote divoire or cote d ivoire or ivory coast or croatia or cuba or cyprus or czech republic or czechoslovakia or djibouti or french somaliland or dominica or dominican republic or ecuador or egypt or united arab republic or el salvador or equatorial guinea or spanish guinea or eritrea or estonia or eswatini or swaziland or ethiopia or fiji or gabon or gabonese republic or gambia or "georgia (republic)" or georgian or ghana or gold coast or gibraltar or greece or grenada or guam or guatemala or guinea or guinea bissau or guyana or british guiana or haiti or hispaniola or honduras or hungary or india or indonesia or timor or iran or iraq or isle of man or jamaica or jordan or kazakhstan or kazakh or kenya or "democratic people’s republic of korea" or republic of korea or north korea or south korea or korea or kosovo or kyrgyzstan or kirghizia or kirgizstan or kyrgyz republic or kirghiz or laos or lao pdr or "lao people's democratic republic" or latvia or lebanon or lebanese republic or lesotho or basutoland or liberia or libya or libyan arab jamahiriya or lithuania or macau or macao or "macedonia (republic)" or macedonia or madagascar or malagasy republic or malawi or nyasaland or malaysia or malay federation or malaya federation or maldives or indian ocean islands or indian ocean or mali or malta or micronesia or federated states of micronesia or kiribati or marshall islands or nauru or northern mariana islands or palau or tuvalu or mauritania or mauritius or mexico or moldova or moldovian or mongolia or montenegro or "montenegro (republic)" or morocco or ifni or mozambique or portuguese east africa or myanmar or burma or namibia or nepal or netherlands antilles or nicaragua or niger or nigeria or oman or muscat or pakistan or panama or papua new guinea or new guinea or paraguay or peru or philippines or philipines or phillipines or phillippines or poland or "polish people's republic" or portugal or portuguese republic or puerto rico or romania or russia or russian federation or ussr or soviet union or union of soviet socialist republics or rwanda or ruanda or samoa or pacific islands or polynesia or samoan islands or navigator island or navigator islands or "sao tome and principe" or saudi arabia or senegal or serbia or seychelles or sierra leone or slovakia or slovak republic or slovenia or melanesia or solomon island or solomon islands or norfolk island or norfolk islands or somalia or south africa or south sudan or sri lanka or ceylon or "saint kitts and nevis" or "st. kitts and nevis" or saint lucia or "st. lucia" or "saint vincent and the grenadines" or saint vincent or "st. vincent" or grenadines or sudan or suriname or surinam or dutch guiana or netherlands guiana or syria or syrian arab republic or tajikistan or tadjikistan or tadzhikistan or tadzhik or tanzania or tanganyika or thailand or siam or timor leste or east timor or togo or togolese republic or tonga or "trinidad and tobago" or trinidad or tobago or tunisia or turkey or "turkey (republic)" or turkmenistan or turkmen or uganda or ukraine or uruguay or uzbekistan or uzbek or vanuatu or new hebrides or venezuela or vietnam or viet nam or middle east or west bank or gaza or palestine or yemen or yugoslavia or zambia or zimbabwe or northern rhodesia or global south or africa south of the sahara or "sub saharan africa" or subsaharan africa or africa, central or central africa or africa, northern or north africa or northern africa or magreb or maghrib or sahara or africa, southern or southern africa or africa, eastern or east africa or eastern africa or africa, western or west africa or western africa or west indies or indian ocean islands or caribbean region or caribbean islands or caribbean or central america or latin america or "south and central america" or south america or asia, central or central asia or asia, northern or north asia or northern asia or asia, southeastern or southeastern asia or south eastern asia or southeast asia or south east asia or asia, western or western asia or europe, eastern or east europe or eastern europe or developing country or developing countries or developing nation? or developing population? or developing world or less developed countr* or less developed nation? or less developed population? or less developed world or lesser developed countr* or lesser developed nation? or lesser developed population? or lesser developed world or under developed countr* or under developed nation? or under developed population? or under developed world or underdeveloped countr* or underdeveloped nation? or underdeveloped population? or underdeveloped world or middle income countr* or middle income nation? or middle income population? or low income countr* or low income nation? or low income population? or lower income countr* or lower income nation? or lower income population? or underserved countr* or underserved nation? or underserved population? or underserved world or under served countr* or under served nation? or under served population? or under served world or deprived countr* or deprived nation? or deprived population? or deprived world or poor countr* or poor nation? or poor population? or poor world or poorer countr* or poorer nation? or poorer population? or poorer world or developing econom* or less developed econom* or lesser developed econom* or under developed econom* or underdeveloped econom* or middle income econom* or low income econom* or lower income econom* or low gdp or low gnp or low gross domestic or low gross national or lower gdp or lower gnp or lower gross domestic or lower gross national or lmic or lmics or third world or lami countr* or transitional countr* or emerging economy or emerging economies or emerging nation?).ti,ab,sh,kw. (2547213)

8 3 and 6 and 7 (5771)

9 limit 8 to exclude medline journals (848)

10 limit 9 to yr="2020 -Current" (362)

| **Author (Year)** | **Country** | **Indicator** | **Effect** | **LCI** | **UCI** | **Effect type** |
| --- | --- | --- | --- | --- | --- | --- |
| Kuwawenaruwa (2019) | Tanzania | Index of drugs available | -0.10 | -0.35 | 0.14 | Regression coefficient |
| Kuwawenaruwa (2019) | Tanzania | Availability of contraceptives | 0.09 | -0.21 | 0.39 | Regression coefficient |
| Kuwawenaruwa (2019) | Tanzania | Medical supplies | -0.01 | -0.26 | 0.25 | Regression coefficient |
| Kuwawenaruwa (2019) | Tanzania | Equipment with problem <90 days | 0.03 | -0.06 | 0.11 | Regression coefficient |
| Kuwawenaruwa (2019) | Tanzania | Electricity function (%) | 0.17 | -0.3 | 0.60 | Regression coefficient |
| Kuwawenaruwa (2019) | Tanzania | Water function (%) | 0.46 | 0.10 | 0.90 | Regression coefficient |
| Kuwawenaruwa (2019) | Tanzania | Toilet function (%) | 0.04 | -0.2 | 0.30 | Regression coefficient |
| Kuwawenaruwa (2019) | Tanzania | Facility quality index (mean) | -0.04 | -0.09 | 0.02 | Regression coefficient |
| Shigute (2020) | Ethiopia | Drug availability | 0.02 | -0.09 | 0.14 | Regression coefficient |
| Shigute (2020) | Ethiopia | Medical equipment/Facility availability | 0.04 | -0.02 | 0.10 | Regression coefficient |
| Shigute (2020) | Ethiopia | Electricity access | 0.06 | -0.25 | 0.36 | Regression coefficient |
| Shigute (2020) | Ethiopia | Water supply | 0.03 | -0.27 | 0.33 | Regression coefficient |
| Shigute (2020) | Ethiopia | Perceived budget shortage | -0.22 | -0.43 | -0.01 | Regression coefficient |
| Shigute (2020) | Ethiopia | Perceived drug shortage | -0.28 | -0.49 | -0.07 | Regression coefficient |

**Appendix 2: Estimates of studies that reported on structural quality of care indicators**

LCI= Lower confidence interval UCI= Upper confidence interval

**Appendix 3: Estimates from studies that reported on processes of care indicators**

| **Author (Year)** | **Country** | **Indicator** | **Effect** | **LCI** | **UCI** | **Effect type** |
| --- | --- | --- | --- | --- | --- | --- |
| **Process-technical** | | | | | | |
| **Content of care** | | | | | | |
| Kuwawenaruwa (2019) | Tanzania | Index of content of care for antenatal care (ANC)-observation of patients | 0.09 | -0.10 | 2.27 | Regression coefficient |
| Kuwawenaruwa (2019) | Tanzania | Index of content of care for ANC-household survey | 0.01 | -0.03 | 0.04 | Regression coefficient |
| Kuwawenaruwa (2019) | Tanzania | Overall postnatal care (PNC) for mothers | 0.18 | 0.06 | 0.30 | Regression coefficient |
| Kuwawenaruwa (2019) | Tanzania | Overall PNC care for infant | 0.19 | -0.11 | 0.48 | Regression coefficient |
| **Process-interpersonal care** | | | | | | |
| **Interpersonal care index** | | | | | | |
| Kuwawenaruwa (2019) | Tanzania | Index of interpersonal care for PNC | 0.24 | 0.03 | 0.46 | Regression coefficient |
| **Waiting time** | | | | | | |
| Kuwawenaruwa (2019) | Tanzania | ANC consultation time-minutes | -3.40 | -13.7 | 6.87 | Regression coefficient |
| Kuwawenaruwa (2019) | Tanzania | PNC consultation time-minutes | 6.05 | -5.93 | 18.0 | Regression coefficient |
| Shigute (2020) | Ethiopia | Waiting time for patient card | -12.8 | -31.8 | 6.09 | Regression coefficient |
| Shigute (2020) | Ethiopia | Waiting time for seeing a doctor/nurse | 0.95 | -0.20 | 0.03 | Regression coefficient |
| **Perceived quality of care** | | | | | | |
| Fink (2013) | Burkina Faso | Facility hours | -0.10 | -0.14 | -0.05 | Regression coefficient |
| Fink (2013) | Burkina Faso | Equipment adequacy | -0.07 | -0.21 | 0.07 | Regression coefficient |
| Fink (2013) | Burkina Faso | Rooms adequacy | -0.04 | -0.18 | 0.09 | Regression coefficient |
| Fink (2013) | Burkina Faso | Drugs available | -0.10 | -0.25 | 0.04 | Regression coefficient |
| Fink (2013) | Burkina Faso | Facility hygiene | -0.20 | -0.38 | -0.01 | Regression coefficient |
| Fink (2013) | Burkina Faso | Staff availability | -0.32 | -0.58 | -0.07 | Regression coefficient |

LCI= Lower confidence interval UCI= Upper confidence interval

**Appendix 4: Estimates from studies that reported on outcome quality of care indicators**

| **Author (Year)** | **Country** | **Indicator** | **Effect** | **LCI** | **UCI** | **Effect type** |
| --- | --- | --- | --- | --- | --- | --- |
| **Patient satisfaction** | | | | | | |
| Shigute (2020) | Ethiopia | Patient satisfaction | 0.18 | 0.30 | 0.06 | Regression coefficient |
| **Self-reported health outcomes** | | | | | | |
| Asuming (2013) | Ghana | Number of days of illness in the last one month | -0.34 | -0.06 | 0.41 | Regression coefficient |
| Asuming (2013) | Ghana | Number of days could not perform normal daily activities due to illness | **-0.81** | **-1.47** | **-0.14** | Regression coefficient |
| Asuming (2013) | Ghana | Could not perform normal daily activities due to illness | -0.03 | -0.09 | 0.03 | Regression coefficient |
| Nguyen (2020) | Vietnam | Number of sick days | -0.22 | -3.29 | 2.86 | Regression coefficient |
| Nguyen (2019) | Vietnam | Number of days staying in bed among children aged 0-2 | -0.11 | -0.26 | 0.15 | Regression coefficient |
| Nguyen (2019) | Vietnam | Number of days staying in bed among children aged 3-5 | 0.02 | -0.22 | 0.28 | Regression coefficient |
| Nguyen (2019) | Vietnam | Number of days with limited activities among children aged 0-2 | -0.15 | -0.58 | 0.28 | Regression coefficient |
| Nguyen (2019) | Vietnam | Number of days with limited activities children aged 3-5 | -0.76 | -1.21 | -0.30 | Regression coefficient |
| Jafree (2021) | Pakistan | Overall perceived good health –nearest neighbor matching | 0.17* | NR | NR | Regression coefficient |
| Sood (2016) | India | Self-care post hospitalization | -0.04 | -0.57 | 0.48 | Regression coefficient |
| Sood (2016) | India | Usual activities-post hospitalization | 0.05 | -0.50 | 0.59 | Regression coefficient |
| Sood (2016) | India | Walking ability-post hospitalization | 0.61 | 0.07 | 1.14 | Regression coefficient |
| Sood (2016) | India | Pain-post hospitalization | 0.56 | 0.08 | 1.04 | Regression coefficient |
| Sood (2016) | India | Anxiety-post hospitalization | 0.39 | -0.15 | 0.92 | Regression coefficient |
| Sood (2016) | India | Overall health post hospitalization | 0.19 | -0.25 | 0.62 | Regression coefficient |
| Sood (2016) | India | Occurrence of infections post operation | -9.40 | -20.2 | 1.40 | Regression coefficient |
| Sood (2016) | India | Been rehospitalised since the first hospitalization | -16.5 | -28.7 | -4.30 | Regression coefficient |
| **Mortality** | | | | | | |
| Fink (2013) | Burkina Faso | Under-five mortality | -1.40 | -8.02 | 5.22 | Regression coefficient |
| Fink (2013) | Burkina Faso | Mortality 65+ | 27.3 | 4.21 | 50.3 | Regression coefficient |
| Lambon-Quayefio (2017) | Ghana | Neonatal mortality | -0.07 | -0.11 | -0.02 | Regression coefficient |
| Philibert (2017) | Mauritania | Neonatal mortality (early days up to 7 days) | 1.67 | 0.74 | 3.8 | Adjusted odds ratio |
| Philibert (2017) | Mauritania | Neonatal mortality (late death of 28 days) | 2.13 | 1.00 | 4.54 | Adjusted odds ratio |
| **Anthropometric measures** | | | | | | |
| Bagnoli (2019) | Ghana | Height-for-age score | 0.17 | 0.09 | 0.25 | Mean difference |
| Nshakira-Rukundo | Uganda | Stunting | -0.04 | -0.08 | -0.00 | Regression coefficient |
| Quimbo (2011) | Philippines | Wasting | -9.0* | NR | NR | Difference in percentage points |
| **Biomarkers** | | | | | | |
| Bagnoli (2019) | Ghana | Not anemic (Hb >100g/l) | 0.10 | 0.07 | 0.14 | Mean Difference |
| Hendriks (2014) | Nigeria | Systolic blood pressure among hypertensive individuals | -5.24 | -9.46 | -1.02 | Regression coefficient |
| Hendriks (2014) | Nigeria | Diastolic blood pressure among hypertensive individuals | -2.16 | -4.27 | -0.05 | Regression coefficient |
| Hendriks (2014) | Nigeria | Controlled hypertension | 3.16 | 0.78 | 12.79 | Regression coefficient |
| Hendriks (2016) | Nigeria | Systolic blood pressure among hypertensive individuals | -4.97 | -10.7 | 0.76 | Regression coefficient |
| Hendriks (2016) | Nigeria | Diastolic blood pressure among hypertensive individuals | -1.81 | -4.68 | 1.06 | Regression coefficient |
| Hendriks (2016) | Nigeria | Controlled hypertension | -0.04 | -0.05 | 0.13 | Regression coefficient |
| Quimbo (2011) | Philippines | CRP positive among children | -4.1* | NR | NR | Difference in percentage points |

LCI= Lower confidence interval UCI= Upper confidence interval NR= Not reported, *Significant at 5% level
